# Quasi‐In Situ 4D‐STEM Mapping of Exsolution Driven Parent Matrix Restructuring in Sr_2_FeMoO_6‐δ_

**DOI:** 10.1002/smtd.70809

**Published:** 2026-07-21

**Authors:** Pritam K. Chakraborty, Junbeom Park, Stephanie E. Wolf, Vaibhav Vibhu, Hongyu Sun, H. Hugo Pérez Garza, Shibabrata Basak, Rüdiger‐A. Eichel

**Affiliations:** ^1^ Institute of Energy Technology‐ Fundamental Electrochemistry (IET‐1) Forschungszentrum Jülich GmbH Jülich Germany; ^2^ Institute of Physical Chemistry RWTH Aachen University Aachen Germany; ^3^ DLR Institut für Technische Thermodynamik Stuttgart Baden‐Württemberg Germany; ^4^ DENSsolutions B.V., Informaticalaan Delft Netherlands; ^5^ Faculty of Mechanical Engineering RWTH Aachen University Aachen Germany

**Keywords:** 4D‐STEM, PFIB‐SEM, quasi‐in situ reduction, solid oxide cells (SOCs)

## Abstract

The in situ exsolution of catalytic particles upon reduction makes double‐perovskites promising candidates for next‐generation solid oxide cells (SOCs). While recent in situ S/TEM studies have –visualized this process, these investigations have primarily focused on isolated particles rather than realistic electrode architectures and the effect of exsolution on the surrounding host matrix remains largely unexplored. Herein, a quasi‐in situ S/TEM approach is presented to investigate the impact of exsolution on a realistic Sr_2_FeMoO_6‐δ_ (SFM) electrode by decoupling the influence of electron‐beam‐induced artifacts. By extracting a lamella from a bulk SOC using a plasma‐FIB and performing identical‐location characterization utilizing EDS and 4D‐STEM before and after reduction captures both the chemical segregation and intrinsic structural transformations within the electrode grains. Utilizing iDPC and radial Fourier analysis (RFA), this approach reveals that surface exsolution is coupled with substantial crystallographic reorganization within the SFM matrix, evidenced by distinct domain shrinkage and localized phase gradients along grain facets. The interconnected bulk electrode geometry promotes higher exsolution density compared to isolated powder particles, underscoring the necessity of studying realistic architectures. By linking surface exsolution to matrix modification in a realistic bulk electrode geometry, this quasi‐in situ methodology provides a vital insights into the exsolution process.

## Graphical abstract

How does catalytic exsolution alter its parent matrix in realistic solid oxide cells? A beam‐artifact‐free, quasi‐in situ 4D‐STEM methodology maps intrinsic nanoscale transformations in bulk Sr_2_FeMoO_6‐δ_ electrodes. Correlative identical‐location imaging reveals that surface exsolution inherently couples with profound crystallographic reorganization and Ruddlesden‐Popper phase formation, offering crucial insights for designing high‐performance energy materials.

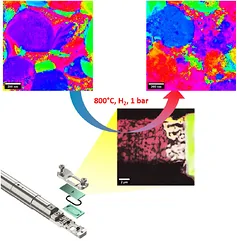

## 1 Introduction

SOCs are high‐temperature (∼600°C–1000°C) electrochemical energy conversion devices that are operated as either solid oxide fuel cells (SOFCs) or solid oxide electrolysis cells (SOECs) [1, 2, 3]. SOCs enable efficient power generation and low‐carbon fuel production, and are posed to play a pivotal role in advancing Power‐to‐X (P*2*X) pathways and supporting global decarbonization targets [4, 5, 6, 7]. Consequently, the long‐term performance, and reliability of SOCs are crucial for their widespread practical deployment. In SOCs, the electrochemical reactions are strongly influenced by the microstructure and catalytic activity of the electrode, and the overall performance of the SOCs is primarily dependent on the electrode performance [8, 9, 10]. While traditional industrial SOCs rely on Ni‐yttria‐stabilized zirconia (Ni‐YSZ) cermets [11, 12, 13, 14], these electrodes suffer from several degradation phenomenon under operating conditions, such as Ni coarsening, carbon coking, redox instability, and sulfur poisoning, reducing the long‐term electrochemical performance of the SOCs [15, 16, 17, 18].

To overcome these limitations, double‐perovskite oxides (A_2_BB′O_6_ structure) have emerged as promising alternative electrode materials. They offer tunable electrochemical properties and demonstrate mixed ionic and electronic conductivity (MIEC) due to the presence of a high concentration of oxygen vacancies [19, 20, 21]. A particularly fascinating property of materials like Sr_2_FeMoO_6‐δ_ (SFM) is the in situ exsolution of catalytic transition metals (e.g., Fe) from the B/B′ cationic sites to the bulk surface upon reduction [21, 22]. These exsolved metallic nanoparticles remain strongly anchored to the parent material surface, significantly enhancing the catalytically active surface area, and thereby improving electrochemical activity and the overall cell efficiency [24, 25, 26]. Furthermore, these strong anchoring suppresses particle coarsening and agglomeration, limits carbon coking, thus addressing the key degradation phenomenon observed for conventional SOC electrodes [27]. In our previous work with SFM‐GDC electrodes, electrolyte‐supported single cells exhibited current densities of –1.26^−^ and –1.27 A cm^−2^ under steam and co‐electrolysis conditions, respectively, outperforming conventional Ni‐YSZ electrodes by ∼38% and approaching Ni‐GDC performance. Long‐term stability was demonstrated over 500 h at –0.3 A cm^−2^ and 900°C under a 50% H_2_O‐50% H_2_ atmosphere [22].

Previous studies elucidated the mechanisms governing B/B′‐site cation exsolution in SFM and its doped variants by reducing isolated powder particles under in situ electron microscopy [22, 28]. While highly informative for visualizing the emergence of nanoparticles, these particle‐based observations fail to capture the complex exsolution mechanisms occurring within interconnected, bulk electrode architectures. Consequently, the structural and crystallographic impact of exsolution on the surrounding parent matrix remains insufficiently resolved. Furthermore, while in situ TEM enables real‐time imaging under controlled temperature, gas, and pressure, continuous electron irradiation at such extreme conditions can induce beam‐induced artefacts that might complicate accurate interpretation of the material's intrinsic behavior [29, 30, 31, 32].

To bridge this methodological gap, we utilized a quasi‐in situ (off‐beam) reduction approach on a bulk SOC lamella. Using a Xe^+^ plasma‐focused ion beam (PFIB), a lamella was extracted from a fully fabricated SOC comprising Sr_2_FeMoO6_−_δ (SFM) electrode, Ce_0.8_Gd_0.2_O_1.9_ (GDC) barrier layer, and 8 mol% yttria‐stabilized zirconia (8YSZ) electrolyte. By reducing the lamella off‐beam in a microelectromechanical systems (MEMS) nanoreactor and performing identical‐location analysis, we eliminate electron‐beam‐driven artifacts while preserving realistic bulk electrode geometry. We map the morphological and chemical changes associated with exsolutions using conventional S/TEM and energy‐dispersive x‐ray spectroscopy (EDS). Crucially, to understand the matrix transformations surrounding the Fe‐exsolutions, we employed 4D‐STEM mapping of the identical regions before and after reduction. This enabled integrated differential phase‐contrast (iDPC) imaging and rapid crystallographic orientation mapping via radial Fourier analysis (RFA) [33, 34, 35, 36]. Although PFIB preparation, MEMS gas‐heating, and correlative imaging are individually established methodologies, the present work combines them to enable a capability that is not available in most exsolution studies, i.e. off‐beam reduction of a SOC‐derived bulk electrode lamella, followed by identical‐location 4D‐STEM mapping to quantify how surface exsolution couples to crystallographic reorganization of the surrounding matrix. This beam‐decoupled, bulk‐relevant approach complements particle‐based quasi‐in situ studies and provides a direct route to separate intrinsic reduction effects from irradiation‐driven artifacts. By coupling off‐beam environmental reduction with 4D‐STEM mapping, this workflow provides a direct link between surface catalytic exsolution with minimized beam‐induced artifacts, and concurrent nanoscale crystallographic reorganization within the parent electrode grains.

## 2 Results and Discussion

To evaluate the microstructural evolution of the bulk electrode, an electron‐transparent cross‐sectional lamella was extracted from a pristine symmetric cell (SFM/GDC/8YSZ/GDC/SFM) using a Xe^+^ PFIB (Figure S1, Table S1). The lamella was then transferred to a gas‐heating MEMS chip (DENSsolutions Climate) and secured with platinum deposition for ensuring mechanical stability. Crucially for this methodology, the chip was mounted in a specialized holder (Climate Infinity) that enables flexible tip orientation, allowing optimized geometries for different electron beam modes.

**FIGURE 1:**
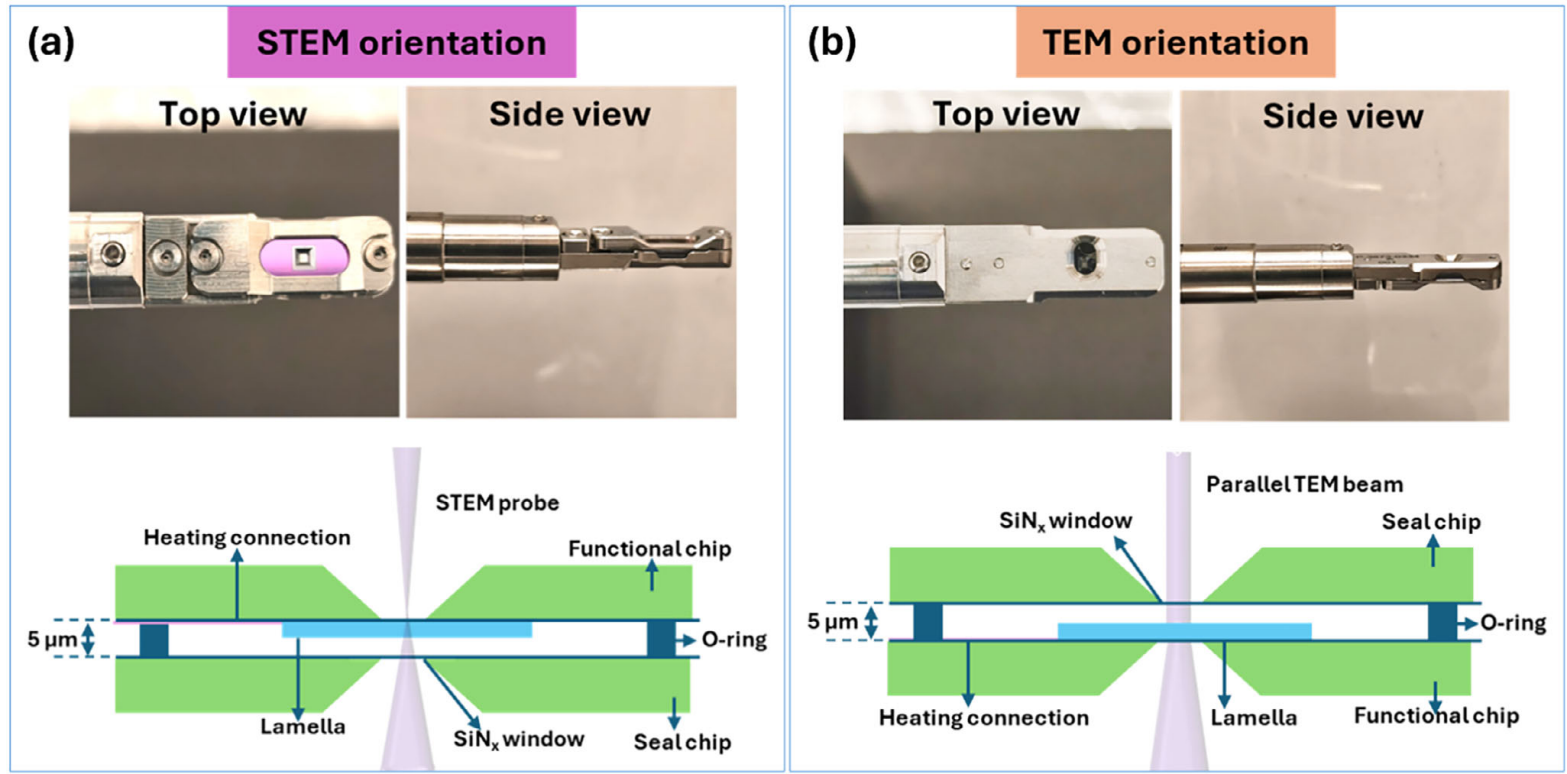
Experimental configuration for quasi‐in situ electron microscopy using a variable‐orientation MEMS holder. (a) Top‐facing configuration used for STEM mode. This arrangement positions the lamella adjacent to the upper SiN_x_ window. (b) Bottom‐facing configuration utilized for TEM mode.

Specifically, for STEM mode operation a top‐facing configuration was used (Figure 1a), positioning the lamella directly beneath the upper electron‐transparent SiN_x_ window. This minimizes the effective gas path before the convergent probe reaches the specimen. This reduces the pre‐sample electron‐beam scattering and preserves the probe coherence, which is critical for achieving the high‐order spatial resolution [37]. In contrast, for TEM imaging mode, a bottom‐facing configuration is preferred (Figure 1b). In this geometry, the parallel beam interacts first with the specimen and subsequently traverses a shorter gas path after interaction. This limits the post‐specimen electron‐beam scattering and thereby maintains the image coherence and high‐resolution information transfer to the microscope camera.

**FIGURE 2:**
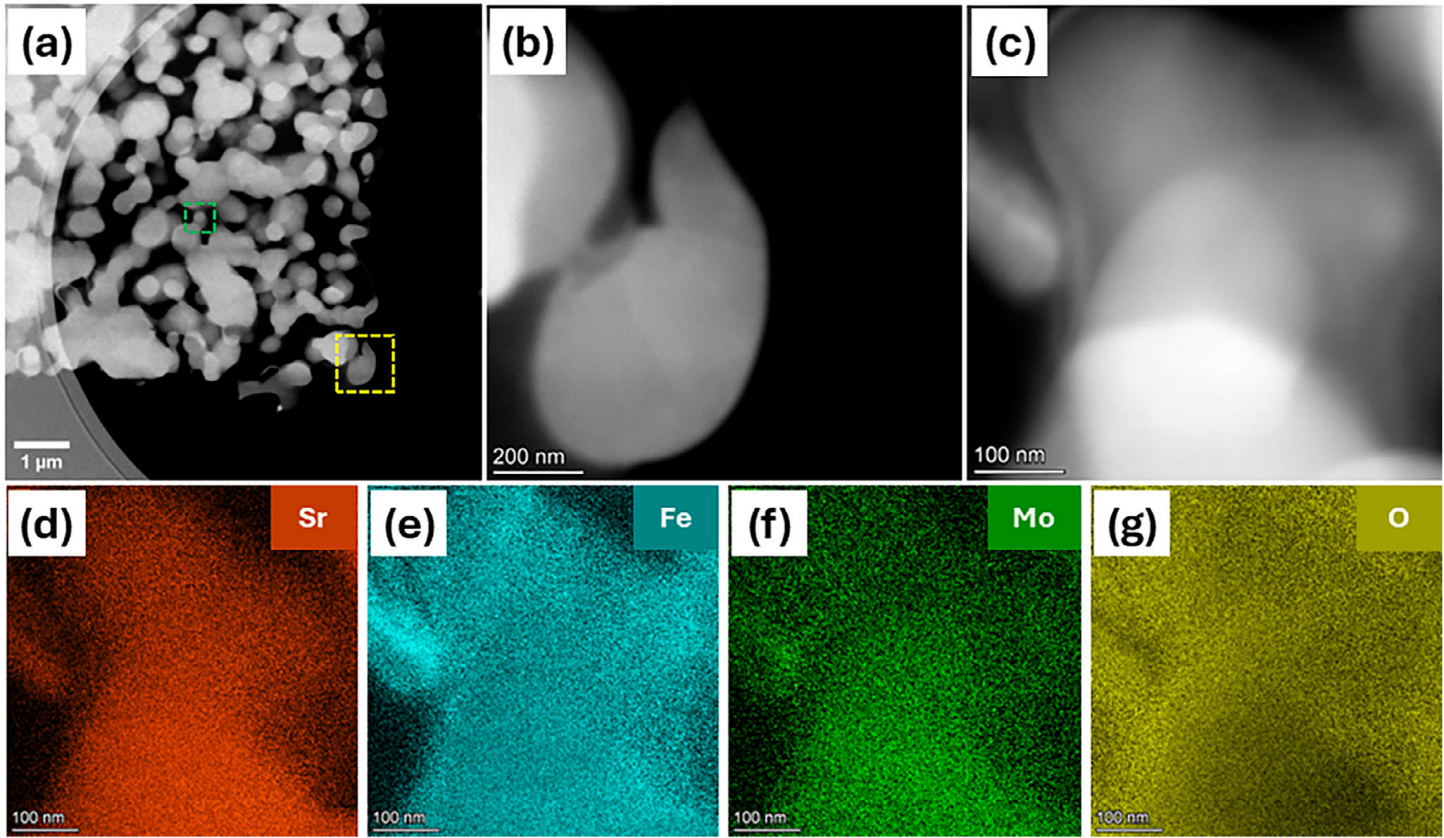
Baseline nanoscale characterization of the as‐prepared SFM bulk electrode lamella. (a) HAADF‐STEM overview image showing SFM electrode of the SOC positioned over the SiN_x_ window. (b,c) Higher magnification HAADF‐STEM images of the highlighted regions (yellow and green boxes in Figure 2a, respectively), showing the smooth, homogeneous surface characteristic of the pristine SFM. (d‐g) EDS map of the corresponding Figure 2c region confirms homogeneous elemental distribution of Sr, Fe, Mo, and O.

Following the assembly in the top‐facing configuration, the electron transparency and structural integrity of the SOC lamella suspended over the SiN_x_ window were first verified by HAADF‐STEM overview imaging (Figure 2a), confirming a continuous, electron‐transparent region suitable for correlative pre‐ and post‐reduction analysis. Higher‐magnification HAADF‐STEM images acquired from representative areas of the SFM electrode, sampled from both the periphery and the centre of the lamella, show a smooth and homogeneous surface without discernible nanoscale secondary features (Figure 2b,c). This appearance is consistent with the pristine, as‐prepared state of SFM and agrees with observations from previous studies [22]. Elemental integrity was further assessed by EDS mapping of the region in Figure 2c, which shows a uniform distribution of Sr, Fe, Mo, and O across the electrode matrix (Figure 2d–g) without evidence of local segregation or milling‐induced compositional artefacts. Together, these results confirm that Xe^+^ ‐PFIB preparation preserved the surface morphology and bulk composition of the SFM matrix, providing a reliable baseline for attributing subsequent microstructural and crystallographic changes to the reduction treatment rather than preparation artefacts.

**FIGURE 3:**
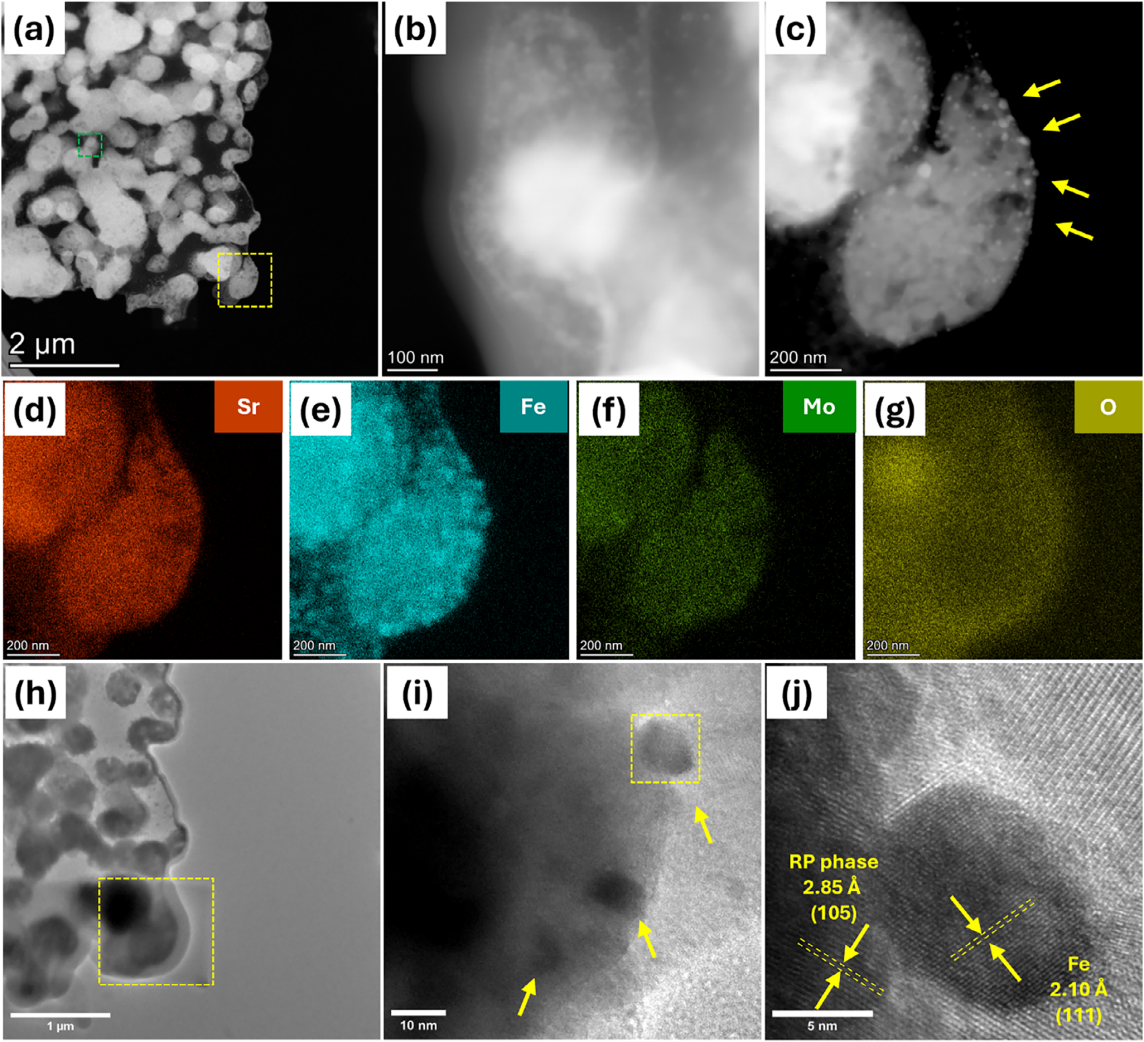
Morphological and chemical evolution of SFM electrode after quasi‐in situ off‐beam reduction (4% H_2_/Ar): (a) Overview image showing reduced SFM electrode. (b, c) High‐magnification HAADF‐STEM images of the highlighted regions (green and yellow boxes in Figure 3a, respectively) showing the high density of exsolved particles. (d‐g) Corresponding EDS elemental maps of the region in (c), confirms the Fe exsolution. (h) BF‐TEM overview image of reduced SFM electrode. (i) High‐magnification BF‐TEM resolving uniform distribution of exsolved particles. (j) HRTEM image resolving lattice parameters of Fe exsolution‐(111) plane, and RP phase‐(105) plane.

To induce exsolution, the lamella was subjected to off‐beam reduction within the MEMS nanoreactor (4% H_2_/Ar at 1 bar, 800°C for 30 min). Subsequent HAADF‐STEM overview imaging of the exact SFM electrode region reveals pronounced surface restructuring, characterized by densification and shrinkage of the electrode (Figure 3a). Higher‐magnification images of the highlighted regions reveal a high‐density of nanoscale surface features (Figure 3b,c). EDS mapping of the area Figure 3c confirms that while the bulk SFM matrix elements remain well‐distributed, discrete, Fe‐enriched nanoparticles have segregated at the surface edges, confirming successful reduction‐driven exsolution [6, 22]. The nano‐exsolutions span 5–45 nm in size, with an average diameter of 19 ± 6 nm (Figure S2). In contrast, the exsolutions in the reduced (100% H_2_ for 10 h at 900°C) bulk SOC of the same composition range between 125–600 nm, with an average diameter of 280 ± 106 nm (Figure S3). The large spread in particle size in the bulk SOC most likely reflects the combined effects of the strongly reducing (100% H_2_) atmosphere and the longer holding time at high temperature. These conditions promote the nucleation and subsequent coarsening of exsolved particles. Furthermore, the availability of the bulk‐electrode network provides abundant diffusion pathways and a larger cation reservoir, enabling sustained material transport for further particle growth. The effect of particle coarsening is also reflected in the exsolution density; the exsolution density per unit area is significantly higher in the reduced SOC lamella (∼350/µm^2^) compared to the reduced bulk SOC (∼3/µm^2^). Aspect‐ratio (AR) analysis (Feret/MinFeret) shows that the lamella exsolutions are largely equiaxed (1.20 ± 0.21; median 1.15), whereas the bulk‐cell exsolutions are more anisotropic (1.54 ± 0.57; median 1.39). The bulk‐cell particles also display a broader distribution with a high‐AR tail extending up to 4.8, indicating a greater prevalence of elongated morphologies in the bulk electrode. The higher anisotropy observed in bulk‐cell exsolutions likely reflects heterogeneous local reduction and extensive network transport conditions within the porous electrode and facet/boundary‐guided growth, with additional contributions from particle coalescence during operation. Notably, when compared to our prior in situ reductions of isolated SFM powder particles, the bulk electrode lamella exhibits a markedly higher density of exsolved features [22]. The higher exsolution density in the lamella likely reflects the continuous, interconnected electrode network, which provides efficient bulk diffusion pathways for Fe transport to the surface.

A complementary bright‐field (BF) TEM image of the reduced SFM electrode is shown in Figure 3h. High‐magnification TEM image (Figure 3i), shows uniform distribution of exsolved nanoparticles on the reduced electrode surface. High‐resolution TEM (HRTEM) confirms the crystallinity of these exsolutions; the measured lattice fringes (d ≈ 2.10 Å) index to the Fe (111) plane, validating the formation of metallic Fe nanoparticles on the surface (Figure 3j). HRTEM analysis further reveals the formation of the layered tetragonal Ruddlesden‐Popper (RP) Sr_3_FeMoO_7_ phase beneath the exsolution; the measured lattice fringes (d ≈ 2.85 Å) indexed to (105) plane. This phase typically forms beneath exsolutions along grain facets of SFM double‐perovskites, which aligns with our previous observations [6].

While formation of surface exsolutions upon reduction is clearly evident, understanding how this reduction‐driven process reshapes the underlying electrode matrix requires deeper crystallographic insight. To assess these changes, we employed 4D‐STEM mapping, acquiring a 2D diffraction pattern (DP) at each scan pixel [35, 38]. This rich 4D‐STEM dataset enables both integrated differential phase‐contrast (iDPC) imaging and radial Fourier analysis (RFA). While the RFA is used for rapid crystallographic orientation mapping, iDPC which is derived via the center‐of‐mass method of deflected beam, provides contrast approximately proportional to the projected electrostatic potential of the specimen. By capturing the beam deflection caused by local electrostatic fields, iDPC offers local phase information, high sensitivity to local structural gradients, and light elements compared to conventional HAADF‐STEM [39, 40, 41, 42, 43, 44].

**FIGURE 4:**
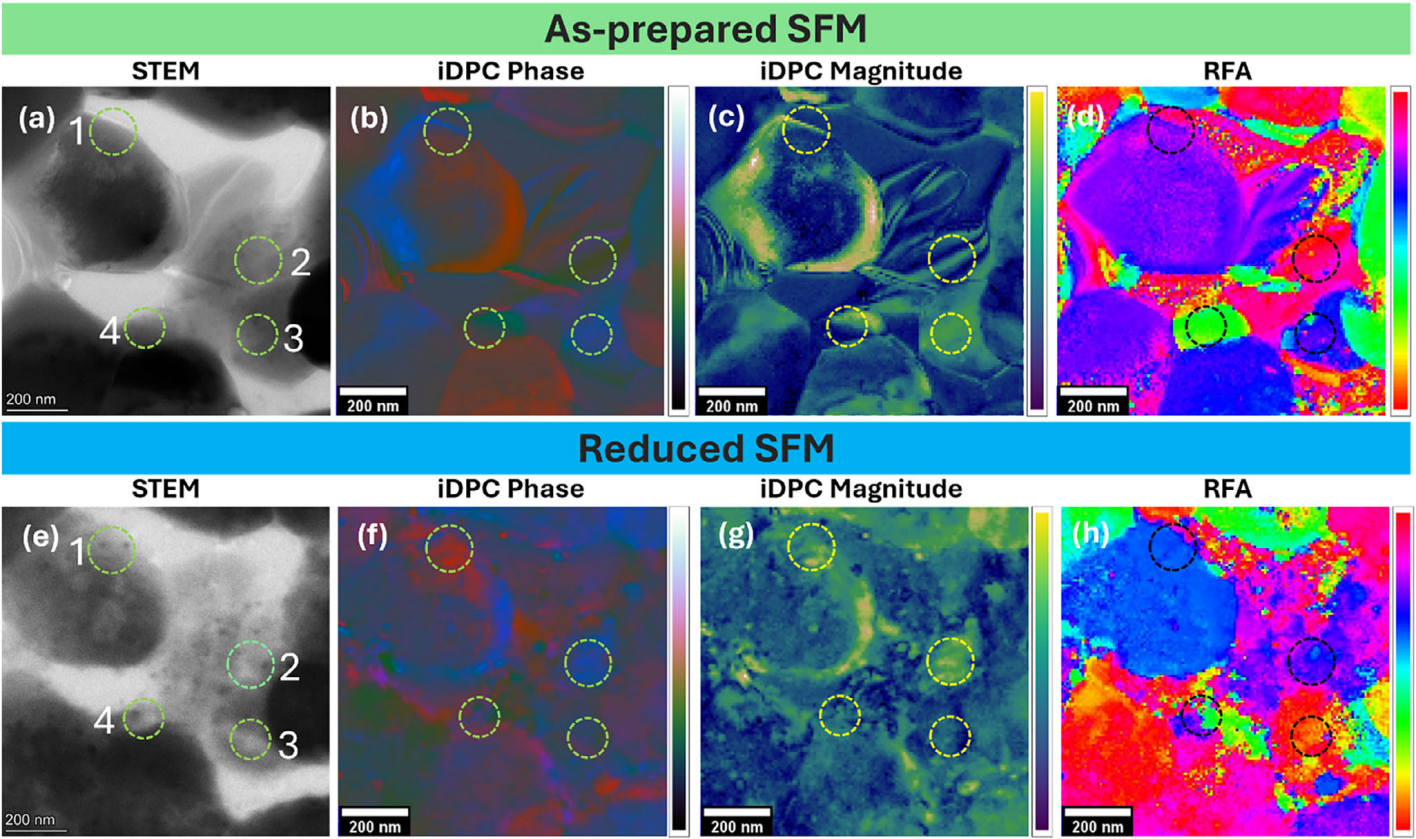
4D‐STEM mapping of crystallographic and phase transformations in SFM in as‐prepared and reduced states. (a, e) Identical‐location HAADF‐STEM images of the selected RoI in the (a) as‐prepared, and (e) reduced states. (b,f) Corresponding iDPC phase maps showing transition from smooth, uniform grain contrast to a state with broader beam deflection and localized phase variations. (c,g) Corresponding iDPC magnitude vector maps revealing enhanced local phase gradients along grain facets after reduction. (d,h)) Reconstructed orientation (angle) map reconstructed with RFA demonstrating that surface exsolution is accompanied by profound bulk domain shrinkage and crystallographic shifts within the identical spatial regions.

For identical‐location analysis, the same region of interest (RoI) was selected for both iDPC and RFA (Figure 4a). In the pre‐reduction (as‐prepared) state, the grains exhibit uniform Z‐contrast in STEM, representative of the overall lamella quality prior to reduction (Figure S4). Correspondingly, the iDPC phase map of the as‐prepared state is relatively smooth at the grain scale (Figure 4b). The iDPC magnitude contrast (Figure 4c) is dominated by grain‐boundary outlines and bend contours rather than strong local potential gradients, a feature consistent with macroscopic lamella bending rather than intrinsic structural heterogeneity [45].

After reduction, the HAADF‐STEM overview of the RoI shows exsolutions at different surfaces at the grain facets (Figure 4e), as shown by the highlighted regions. After reduction, the bend contours at grain boundaries are no longer observed, consistent with stress relaxation within the grains due to high‐temperature annealing. It is observable from the iDPC phase and magnitude vector maps that the regions exhibit smaller localized variations in comparison to the as‐prepared SFM grains (Figure 4f,g), indicating a broader spread of deflection directions and stronger local phase gradients. Deflection was quantified from the vector CoM conversion of the 4D‐STEM dataset, showing markedly higher beam‐deflection magnitude in exsolution‐rich regions, particularly near the grain edges, than in central grain regions. Bright regions around grain edges (most evident near the highlighted sites 1 and 2), indicate that strong deflection extends laterally along grain facets rather than being confined to discrete exsolution points (Figure 4g). This strong deflection along the grain facets is most likely due to the formation of an underlying layered tetragonal RP phase. RP layering can support distinct oxygen‐defect configurations (vacancies and interstitial oxygen) and modified oxygen transport pathways, which may contribute to the strong local phase‐gradient/deflection signatures measured near facet regions after reduction [46].

Similar high‐contrast regions along grain facets, coinciding with higher exsolution density, are also observed in other reduced areas analyzed by iDPC (Figure S5). The weaker iDPC contrast and smoother phase in the central regions of grains are consistent with their greater local thickness, leading to increased multiple scattering events, which can partially mask exsolution‐related signals. It needs to be stressed that iDPC contrast at this magnification is prone to thickness variation, dynamical scattering, and local bending. Thus, to eliminate the possibility that the generated iDPC contrast is merely an artifact of lower local thickness around the grain edges, and to confirm it is driven by the newly formed RP phase, it is critical to obtain local crystallographic information from these regions. To validate these crystallographic transformations, the same RoI was investigated using RFA. RFA reconstructs orientation maps by extracting the dominant angular distribution (∼0‐360°) from the pixelated DPs [33]. These angular distributions from the RoI were then used to generate the orientation (angle) maps (Figure 4d,h) before and after reduction, respectively. The orientation map of the as‐prepared SFM lamella resolves distinct, coarse‐grained orientations that closely follow the grain morphology observed in the corresponding HAADF‐STEM image (Figure 4d). The reconstructed electron DPs from the highlighted regions (1‐4) in the RoI exhibit orientations consistent with cubic Sr(Fe_0.8_Mo_0.2_)O_3_ (ICSD‐191551) and tetragonal SrMoO_4_ (ICSD‐99089) phases (Figure S6). These are the same phases present in as‐prepared SFM before reduction, as proven in our earlier investigation [22]. The RFA orientation color map of the same RoI exhibits drastic orientation changes and domain shrinkages within the same angular distribution range after reduction (Figure 4h). Such changes indicate that reduction‐driven exsolution is accompanied by crystallographic rearrangement within the SFM grains rather than a purely surface‐localized process. Comparison of the reconstructed electron DPs from the same highlighted regions (1‐4) of the reduced SFM RoI, exhibit orientations consistent with reduction‐driven lattice reorganization and partial phase evolution of SFM toward Sr_2_(Fe_1.33_Mo_0.66_)O_5.88_ (ICSD‐168704), and the tetragonal Sr_3_FeMoO_7_ (RP phase) (ICSD‐156787) phases, both previously linked to Fe exsolution in reduced SFM (Figure S6) [21, 22, 23]. The formation of the RP phase in the highlighted regions 1 and 2 of the RoI, provides a plausible basis for the stronger deflection contrast observed around the grain boundaries in the iDPC color‐map of the reduced SFM. Similar domain shrinkages and reorientations were observed at multiple locations within identical angular thresholds across the SFM electrode after reduction, further supporting this observation (Figure S5).

Similar to Co‐doped SFM systems where reduction induces a coupled transformation into an RP phase plus metallic alloy nanoparticles, the facet‐localized contrast and domain redistribution observed here are consistent with an RP‐type near‐surface transformation accompanying exsolution [46]. This observation indicates that the pronounced domain reorganization in the present study is dominated by the chemically driven redox processes (oxygen exchange, vacancy formation, and associated lattice reorganization) that accompany reduction.

Exsolution process is commonly described as a sequence of surface cation diffusion, reduction, nucleation, and growth; the present correlative 4D‐STEM results extend this picture by capturing the accompanying crystallographic response of the host matrix in a bulk electrode geometry. By combining conventional STEM, TEM, and EDS with advanced 4D‐STEM techniques (iDPC and RFA), this approach delivers a comprehensive, artifact‐free view of how surface catalytic exsolution inherently couples to parent matrix reorganization in a realistic bulk geometry. Overall, the off‐beam quasi‐in situ workflow seamlessly correlates surface Fe‐exsolutions with concurrent microstructural and crystallographic changes in the SFM electrode.

## 3 Conclusion

In this study, we demonstrated a robust, off‐beam quasi‐in situ electron microscopy workflow to investigate the reduction‐driven exsolution of Sr_2_FeMoO_6‐δ_ within a realistic solid oxide cell geometry. By extracting a pristine SFM lamella and performing identical‐location 4D‐STEM analysis before and after reduction in a MEMS reactor, we successfully isolated the intrinsic, thermodynamically driven material changes while eliminating the electron‐beam‐induced artifacts that frequently complicate in situ TEM data interpretations. Conventional STEM, TEM, and EDS confirmed that exsolution is highly active within the interconnected electrode network, yielding a denser array of exsolved Fe nanoparticles compared to isolated powder particles. Crucially, the application of advanced 4D‐STEM techniques, specifically iDPC and RFA, revealed that surface exsolution is intimately linked to profound nanoscale reorganization within the bulk parent matrix. While iDPC visualized extended phase gradients indicative of Ruddlesden‐Popper phase formation along the grain facets, RFA provided direct crystallographic evidence of concurrent domain shrinkage and phase evolution. Beyond validating exsolution trends in double‐perovskites, this identical‐location methodology establishes a direct, artifact‐free link between catalytic surface phenomena and bulk matrix structural responses. The datasets generated by this approach can be instrumental in identifying microstructural motifs that promote controlled exsolution, diagnosing degradation‐induced phase transformations, and ultimately guiding the rational design of more stable, high‐performance electrodes for next‐generation SOCs. This method can also be further extended to other energy conversion systems such as battery electrodes, catalysts, redox oxides for deeper‐dive into understanding their structural transformations in different environments under external stimuli of temperature and bias.

## 4 Experimental section

### 4.1 Material Synthesis and Cell Fabrication

Sr_2_FeMoO_6‐δ_ (SFM) powder was synthesized via a conventional solid‐state route using precursor powders of MoO_3_ (Alfa Aesar, 99%), SrCO_3_ (Aldrich Chem, 99%), and Fe_2_O_3_ (Alfa Aesar, 99%) as previously established [22]. Commercial gadolinium‐doped ceria (GDC, Ce_0.8_Gd_0.2_O_1.9_, SOFCMAN, 99.5%) was used as a buffer layer between the electrodes and the 8 mol% yttria‐stabilized zirconia (8YSZ) electrolyte. Each powder (SFM, and GDC) was mixed at a 50:50 wt.% ratio with a slurry solution of 3 wt.% ethyl cellulose (binder) in α‐terpineol (dispersant). The slurries were homogenized using a planetary vacuum mixer (THINKY Mixer ARV‐310, C3 Prozess und Analysentechnik GmbH, Germany) followed by roll milling. Single cells were fabricated by sequential screen printing (EKRA Screen Printing Technologies) on commercially available 8YSZ dense substrates (Kerafol, Germany; ∼20 mm diameter, ∼250 µm thickness). First, GDC buffer layers (∼5‐7 µm thick, ∼18 mm diameter) were printed on both sides and sintered at 1375°C for 1 h in air to prevent the formation of insulating interfacial phases. Subsequently, symmetrical electrodes of SFM (∼15‐20 µm thick, ∼10 mm diameter) were printed on both sides and sintered at 1150°C for 2 h in air, yielding symmetrical SFM/GDC/8YSZ/GDC/SFM cells with homogeneous microstructures. The fabricated cells were fractured, embedded in epoxy, and polished to expose flat cross‐sectional surfaces suitable for PFIB lamella preparation.

### 4.2 Lamella Preparation

Lamella preparation was conducted on Tescan AmberX Plasma FIB‐SEM. SE and BSE images were acquired at 5 kV and 300 pA, and EDS maps at 20 kV and 1 nA. PFIB parameters for lamella preparation and MEMS‐transfer are provided within supplementary information.

### 4.3 Quasi‐In Situ Reduction

Reduction was performed on the SOC lamella mounted on DENSsolutions Climate gas‐heating MEMS chip in a Climate Infinity holder. Off‐beam reduction used 4% H_2_/Ar at 1 bar and 1 mL/min flow rate, for 30 min at 800°C. Prior to reduction, the assembled MEMS chamber within the holder was purged with Ar (5 mL/min) at 300°C for 15 min to minimize contamination.

### 4.4 STEM & TEM Characterization

Bright field (BF), and high‐angle annular dark field (HAADF) STEM images were acquired on a Cs probe‐corrected FEI Titan G2 80–200 ChemiSTEM operated at 200 kV. EDS maps were collected using the Super‐X EDX detector (ChemiSTEM). BF‐TEM imaging was performed on an FEI Titan 80–300 kV TEM. 4D‐STEM data was collected using a pixelated 4D STEM detector (EMPAD), and the data analysis was performed by considering a virtual ring detector via python based LiberTEM packages for RFA, and iDPC methods [47].

## Conflicts of Interest

The authors declare no conflicts of interest.

## Supporting information




**Supporting File**: smtd70809‐sup‐0001‐SuppMat.docx.

## Data Availability

The data that support the findings of this study are available from the corresponding author upon reasonable request.

## References

[1] H. J. Lee , S. J. Son , S. K. Kim , et al., “A‐Site and B‐Site Co‐Doped PrBaFe_2_O_5+δ_ Double Perovskite as a Symmetrical Electrode of Solid Oxide Fuel Cell With Potential of CO_2_ Electrolysis,” Journal of Power Sources 595 (2024): 234032, 10.1016/j.jpowsour.2023.234032.

[2] A. B. Muñoz‐García , M. Pavone , and E. A. Carter , “Effect of Antisite Defects on the Formation of Oxygen Vacancies in Sr_2_FeMoO_6_: Implications for Ion and Electron Transport,” Chemistry of Materials 23, no. 20 (2011): 4525–4536.

[3] Q. Xu , Z. Guo , L. Xia , et al., “A comprehensive Review of Solid Oxide Fuel Cells Operating on Various Promising Alternative Fuels,” Energy Conversion and Management 253 (2022): 115175, 10.1016/j.enconman.2021.115175.

[4] T. Chen , W. Guan , C. Ma , et al., “Highly Efficient Direct Carbon Solid Oxide Fuel Cells Operated With Camellia Oleifera Biomass,” Electrochimica Acta 423 (2022): 140594, 10.1016/j.electacta.2022.140594.

[5] P. Chakraborty , L. de Haart , S. Basak , E. Jodat , A. Karl , and R.‐A. Eichel , “In Optimization of Solid Oxide Fuel Cell materials: Visualization of Processes Via In‐Situ Electron Microscopy,” IET1 PHD seminar (No. FZJ‐2025‐00672). Grundlagen der Elektrochemie (2024).

[6] P. K. Chakraborty , S. E. Wolf , G. Ummethala , et al., “Unveiling the Exsolution Mechanisms and Investigation of the Catalytic Processes of Sr_2_FeMo_0.65_Ni_0.35_O_6‐δ_ Using In Situ Transmission Electron Microscopy,” Nano Today 61 (2025): 102649, 10.1016/j.nantod.2025.102649.

[7] Z. Ma , W. L. Dacayan , C. Chatzichristodoulou , et al., “Electrochemical Impedance Spectroscopy Integrated With Environmental Transmission Electron Microscopy,” Small Methods 7, no. 7 (2023): 2201713, 10.1002/smtd.202201713. 37035947

[8] C. Graves , S. D. Ebbesen , S. H. Jensen , S. B. Simonsen , and M. B. Mogensen , “Eliminating Degradation in Solid Oxide Electrochemical Cells by Reversible Operation,” Nature materials 14, no. 2 (2015): 239–244, 10.1038/nmat4165. 25532070

[9] J. Zhang , M. Barreau , T. Dintzer , et al., “Unveiling Key Interface Characteristics of Ni/Yttria‐Stabilized Zirconia Solid Oxide Cell Electrodes in H_2_O Electroreduction Using Operando X‐ray Photoelectron Spectroscopy,” ACS Applied Materials & Interfaces 16, no. 29 (2024): 37915–37926, 10.1021/acsami.4c05046. 38989828

[10] J. Lee , T. Kim , H. J. Lee , K. J. Yoon , J.‐H. Lee , and J. H. Joo , “Understanding the Degradation Mechanisms of Ba‐Based Double Perovskite Electrodes for Solid Oxide Electrochemical Cells,” Chemical Engineering Journal 505 (2025): 159654, 10.1016/j.cej.2025.159654.

[11] Y. Song , Z. Zhou , X. Zhang , et al., “Pure CO_2_ Electrolysis Over an Ni/YSZ Cathode in a Solid Oxide Electrolysis Cell,” Journal of Materials Chemistry A 6, no. 28 (2018): 13661–13667, 10.1039/C8TA02858C.

[12] M. Pihlatie , A. Kaiser , and M. Mogensen , “Mechanical Properties of NiO/Ni–YSZ Composites Depending on Temperature, Porosity and Redox Cycling,” Journal of the European Ceramic Society 29, no. 9 (2009): 1657–1664, 10.1016/j.jeurceramsoc.2008.10.017.

[13] Z. Zhan and L. Zhao , “Electrochemical Reduction of CO_2_ in Solid Oxide Electrolysis Cells,” Journal of Power Sources 195, no. 21 (2010): 7250–7254, 10.1016/j.jpowsour.2010.05.037.

[14] E. Ghosh , A. Akter , J. R. Mulligan , S. Gopalan , U. B. Pal , and S. N. Basu , “Low‐Voltage SEM Imaging of the Four‐Phase Microstructure of the Fuel Electrode of a Solid Oxide Electrolysis Cell Using Mixed Detector Signals to Quantify Location Specific Percolated Ni Loss,” International Journal of Hydrogen Energy 202 (2026): 153111, 10.1016/j.ijhydene.2025.153111.

[15] J. Feng , G. Yang , N. Dai , et al., “Investigation Into the Effect of Fe‐Site Substitution on the Performance of Sr_2_Fe_1.5_Mo_0.5_O_6− δ_ Anodes for SOFCs,” Journal of Materials Chemistry A 2, no. 41 (2014): 17628–17634.

[16] K. Zhu , T. Wu , M. Li , R. Lu , X. Zhu , and W. Yang , “Perovskites Decorated With Oxygen Vacancies and Fe–Ni Alloy Nanoparticles as High‐Efficiency Electrocatalysts for the Oxygen Evolution Reaction,” Journal of Materials Chemistry A 5, no. 37 (2017): 19836–19845, 10.1039/C7TA05404A.

[17] Y. Gao , D. Chen , M. Saccoccio , Z. Lu , and F. Ciucci , “From Material Design to Mechanism Study: Nanoscale Ni Exsolution on A Highly Active A‐Site Deficient Anode Material for Solid Oxide Fuel Cells,” Nano Energy 27 (2016): 499–508, 10.1016/j.nanoen.2016.07.013.

[18] Z. Du , H. Zhao , S. Yi , et al., “High‐Performance Anode Material Sr_2_FeMo_0.65_Ni_0.35_O_6−δ_ With In Situ Exsolved Nanoparticle Catalyst,” ACS nano 10, no. 9 (2016): 8660–8669, 10.1021/acsnano.6b03979. 27529355

[19] Y.‐H. Huang , R. I. Dass , Z.‐L. Xing , and J. B. Goodenough , “Double Perovskites as Anode Materials for Solid‐Oxide Fuel Cells,” Science 312, no. 5771 (2006): 254–257, 10.1126/science.1125877. 16614219

[20] N. Dai , J. Feng , Z. Wang , et al., “Synthesis and Characterization of B‐Site Ni‐Doped Perovskites Sr_2_Fe_1.5−x_Ni_x_Mo_0.5_O_6−δ_(x = 0, 0.05, 0.1, 0.2, 0.4) as Cathodes for SOFCs,” Journal of Materials Chemistry A 1, no. 45 (2013): 14147–14153, 10.1039/c3ta13607h.

[21] Q. Liu , X. Dong , G. Xiao , F. Zhao , and F. Chen , “A Novel Electrode Material for Symmetrical SOFCs,” Advanced materials 22, no. 48 (2010): 5478–5482, 10.1002/adma.201001044. 20941791

[22] S. E. Wolf , V. Vibhu , P. K. Chakraborty , et al., “Electrochemical Performance and Durability of High‐temperature Solid Oxide Electrolysis Cells with SFM and SFM‐GDC Fuel Electrodes for Hydrogen and Syngas Production,” Journal of Materials Chemistry A 13, no. 40 (2025): 34565–34584.

[23] S. E. Wolf , M. Wessling , J. Mayer , and R.‐A. Eichel , “Electrochemical Analysis and Development of Novel Electrode Materials for High‐Temperature Electrolysis,” Lehrstuhl für Materialien und Prozesse für elektrochemische Energiespeicher (2025), 51.

[24] H. Lv , L. Lin , X. Zhang , et al., “In Situ Investigation of Reversible Exsolution/Dissolution of CoFe Alloy Nanoparticles in a Co‐Doped Sr_2_Fe_1.5_Mo_0.5_O_6−δ_ Cathode for CO_2_ Electrolysis,” Advanced Materials 32, no. 6 (2020): 1906193, 10.1002/adma.201906193. 31894628

[25] Y. Liao , X. Xi , H. Chen , J. Liu , X.‐Z. Fu , and J.‐L. Luo , “The Emerging Sr_2_FeMoO_6_‐based Electrocatalysts for Solid Oxide Electrochemical Cell: Synthesis, Modulation and Applications,” Chemical Synthesis 4, no. 2 (2024): 3.

[26] H. Sun , X. Xu , Y. Song , and Z. Shao , “Recent Progress in Sr_2_Fe_1.5_Mo_0.5_O_6‐δ ‐_Based Multifunctional Materials for Energy Conversion and Storage,” Advanced Functional Materials 34, no. 51 (2024): 2411622, 10.1002/adfm.202411622.

[27] M. E. Ibrahim , A. Mustafa , S. Le , et al., “A Critical Review on the Design Strategies of SFM‐Based Perovskite Oxides for High‐Temperature CO_2_ Electrolysis in Solid Oxide Electrolysis Cells,” Sustainable Energy & Fuels 10 (2026): 405–446 .

[28] H. Zheng , P. K. Chakraborty , R. Spruit , et al., “Modulating Metal Support Interactions for Durable and Selective CO_2_ Hydrogenation Reaction: An Operando Transmission Electron Microscopy Study,” Microscopy and Microanalysis 31 (2025): ozaf048.

[29] W. Bruenger , H. Kleinschmidt , W. Hässler‐Grohne , and H. Bosse , “Contamination Reduction in Low Voltage Electron‐Beam Microscopy for Dimensional Metrology,” Journal of Vacuum Science & Technology B: Microelectronics and Nanometer Structures Processing, Measurement, and Phenomena, Measurement, and Phenomena 15, no. 6 (1997): 2181–2184, 10.1116/1.589349.

[30] N. Jiang , “Electron Beam Damage in Oxides: A Review,” Reports on Progress in Physics 79, no. 1 (2015): 016501, 10.1088/0034-4885/79/1/016501. 26684361

[31] M. Hugenschmidt , K. Adrion , A. Marx , E. Müller , and D. Gerthsen , “Electron‐Beam‐Induced Carbon Contamination in STEM‐in‐SEM: Quantification and Mitigation,” Microscopy and Microanalysis 29, no. 1 (2023): 219–234, 10.1093/micmic/ozac003.

[32] S. Basak , J. Jansen , Y. Kabiri , and H. W. Zandbergen , “Towards Optimization of Experimental Parameters for Studying Li‐O_2_ Battery Discharge Products in TEM Using In Situ EELS,” Ultramicroscopy 188 (2018): 52–58, 10.1016/j.ultramic.2018.03.005. 29554486

[33] J. Park , S. Dutta , H. Sun , et al., “Toward Quantitative Electrodeposition via In Situ Liquid Phase Transmission Electron Microscopy: Studying Electroplated Zinc Using Basic Image Processing and 4D STEM,” Small Methods 8, no. 12 (2024): 2400081, 10.1002/smtd.202400081. 38686691 PMC11672167

[34] C. Ophus , “Four‐Dimensional Scanning Transmission Electron Microscopy (4D‐STEM): From Scanning Nanodiffraction to Ptychography and Beyond,” Microscopy and Microanalysis 25, no. 3 (2019): 563–582, 10.1017/S1431927619000497. 31084643

[35] M. Zhu , J. Lanier , J. Flores , et al., “Structural Degeneracy and Formation of Crystallographic Domains in Epitaxial LaFeO_3_ Films Revealed by Machine‐Learning Assisted 4D‐STEM,” Scientific Reports 14, no. 1 (2024): 4198, 10.1038/s41598-024-54661-1. 38378717 PMC10879141

[36] A. Clausen , D. Weber , M. Bryan , et al., LiberTEM/LiberTEM: 0.12.0, (2024), 10.5281/ZENODO.8252548.

[37] M. L. San Gabriel , C. Qiu , D. Yu , T. Yaguchi , and J. Y. Howe , “Simultaneous Secondary Electron Microscopy in the Scanning Transmission Electron Microscope With Applications for In Situ Studies,” Microscopy 73, no. 2 (2024): 169–183, 10.1093/jmicro/dfae007. 38334743

[38] Y. Ding , Y. Choi , Y. Chen , K. C. Pradel , M. Liu , and Z. L. Wang , “Quantitative Nanoscale Tracking of Oxygen Vacancy Diffusion Inside Single Ceria Grains by in situ transmission electron microscopy,” Materials Today 38 (2020): 24–34, 10.1016/j.mattod.2020.04.006.

[39] I. Lazić , E. G. Bosch , and S. Lazar , “Phase Contrast STEM for Thin Samples: Integrated Differential Phase Contrast,” Ultramicroscopy 160 (2016): 265–280. 26590505 10.1016/j.ultramic.2015.10.011

[40] N. Shibata , T. Seki , G. Sánchez‐Santolino , et al., “Electric Field Imaging of Single Atoms,” Nature communications 8, no. 1 (2017): 15631, 10.1038/ncomms15631. PMC5459997 28555629

[41] G. Sánchez‐Santolino , N. R. Lugg , T. Seki , et al., “Probing the Internal Atomic Charge Density Distributions in Real Space,” ACS nano 12, no. 9 (2018): 8875–8881, 10.1021/acsnano.8b03712. 30074756

[42] N. H. Dekkers and H. de Lang “Differential phase contrast in a STEM,”, Optik 41, no. 4 (1974): 452–456.

[43] Z. Liang , D. Song , and B. Ge , “Optimizing Experimental Parameters of Integrated Differential Phase Contrast (iDPC) for Atomic Resolution Imaging,” Ultramicroscopy 246 (2023): 113686, 10.1016/j.ultramic.2023.113686. 36682324

[44] N. Shibata , S. D. Findlay , H. Sasaki , et al., “Imaging Of Built‐In Electric Field at a p‐n Junction by Scanning Transmission Electron Microscopy,” Scientific reports 5, no. 1 (2015): 10040, 10.1038/srep10040. 26067359 PMC4464396

[45] Y. O. Murakami , T. Seki , A. Kinoshita , T. Shoji , Y. Ikuhara , and N. Shibata , “Magnetic‐Structure Imaging in Polycrystalline Materials by Specimen‐Tilt Series Averaged DPC STEM,” Microscopy 69, no. 5 (2020): 312–320, 10.1093/jmicro/dfaa029. 32455425

[46] Y. Li , M. Singh , Z. Zhuang , et al., “Efficient Reversible CO/CO_2_ Conversion in Solid Oxide Cells With a Phase‐Transformed Fuel Electrode,” Science China Materials 64, no. 5 (2021): 1114–1126, 10.1007/s40843-020-1531-7.

[47] A. Clausen , D. Weber , K. Ruzaeva , et al., “LiberTEM: Software Platform for Scalable Multidimensional Data Processing in Transmission Electron Microscopy,” Journal of Open Source Software 5, no. 50 (2020): 2006, 10.21105/joss.02006.

