# Supplementary material for: Quasi‐In Situ 4D‐STEM Mapping of Exsolution Driven Parent Matrix Restructuring in Sr2FeMoO6‐δ
Source: Small Methods. 2026 Jul 21;10(16):e70809. doi: 10.1002/smtd.70809 (PMC13505689; doi:10.1002/smtd.70809)
Supplement: Supplementary file 1 [file SMTD-10-e70809-s001.docx]

**Quasi-in situ 4D-STEM mapping of exsolution driven parent matrix restructuring in** **Sr_2_FeMoO_6‑δ_**

Pritam K. Chakraborty^1,2^, Junbeom Park^1^, Stephanie E. Wolf^1,3^, Vaibhav Vibhu^1^, Hongyu Sun^4^, Hugo Pérez Garza^4^, Shibabrata Basak^1*^, Rüdiger-A Eichel^1,2^

^1^Institute of Energy Technology- Fundamental Electrochemistry (IET-1),

Forschungszentrum Jülich GmbH, 52425 Jülich, Germany.

^2^ Institute of Physical Chemistry, RWTH Aachen University, Aachen, Germany.

^3^ DLR Institut für Technische Thermodynamik, Stuttgart, Baden-Württemberg, Germany.

^4^ DENSsolutions B.V., Informaticalaan 12, 2628 ZD Delft, Netherlands.

^5^ Faculty of Mechanical Engineering, RWTH Aachen University, 52056 Aachen, Germany.

** Corresponding author:* *[s.basak@fz-juelich.de](C:\\Users\\p.chakraborty\\Desktop\\PhD\\Papers\\SFM paper (lamella)\\Final reviewer comment inc\\s.basak@fz-juelich.de)*

**Figure S1.** (a-h) SE and BSE images of different stages of SOC lamella preparation and transfer to MEMS. (i) EDS map of the as-prepared SOC lamella showing the main phases of the electrode, barrier layer, and electrolyte.

| **Preparation steps** | **Dimensions** | **Stage-tilt** | **Stage rotation** | **PIB Current** | **PIB Voltage** |
| --- | --- | --- | --- | --- | --- |
| Platinum protection layer | 25 µm × 3 µm × 5 µm | 55° | 0° | 100 pA | 30 kV |
| Trench milling | 35 µm × 15 µm × 20 µm | 55° | 0° | 5 nA | 30 kV |
| Trench polishing | 35 µm × 15 µm × 20 µm | 55° | 0° | 1 nA | 30 kV |
| Undercut | *b* = 1 µm, *t* = 3 µm | 0° | 0° | 1 nA | 30 kV |
| Nano-manipulator attachment | Pt weld thickness = 1 µm | 55° | 0° | 50 pA | 30 kV |
| Bridge-cut | *t* = 1 µm | 55° | 0° | 1 nA | 30 kV |
| Lamella lift-out | -- | 55° | 0° | -- | 30 kV |
| Half-grid attachment | Pt weld thickness = 0.5 µm | 55° | 0° | 50 pA | 30 kV |
| Initial Lamella thinning | ~ 150 nm | 57° | 0°, 180° | 100, 50, 20 pA | 30 kV |
| Final Lamella thinning | ~ 120 nm | 57° | 0°, 180° | 20 pA | 10 kV |

Supporting Table T1: Fabrication steps and corresponding parameters for SOC lamella preparation.

**Figure S2.** (a) Selected area from HAADF-STEM image of reduced (4% H_2_/Ar at 1 bar, 800 °C for 30 min) SOC lamella for particle size analysis. (b) Digitally inverted BF image of the selected area for particle size analysis. (c) Particle size distribution. (d) Particle area distribution.

**Figure S3.** (a) SEM (BSE) image of a reduced (100% H_2_ for 10 hours at 800 °C) bulk SOC for particle size analysis. (b) Segmented image obtained after thresholding. (c) Outlines of the Fe nano-exsolutions. (d) Particle size distribution. (e) Particle area distribution.

**Figure S4.** (a,e) HAADF-STEM images of different RoIs in as-prepared SFM; (b,f) iDPC phase maps of the ROIs in as-prepared SFM; (c,g) iDPC magnitude maps of the ROIs in as-prepared SFM; (d,h) ) Reconstructed orientation (angle) maps from second-order spatial frequencies from 4D-STEM DPs of as-prepared SFM.

**Figure S5.** (a,e) HAADF-STEM images of different RoIs in reduced SFM; (b,f) iDPC phase maps of the ROIs in reduced SFM; (c,g) iDPC magnitude maps of the ROIs in reduced SFM; (d,h) ) Reconstructed orientation (angle) maps from second-order spatial frequencies from 4D-STEM DPs of reduced SFM.

**Figure S6.** Comparison of reconstructed electron DPs from as-prepared SFM SOC lamella at different highlighted regions (1-4) with simulated electron DP using cubic Sr(Fe_0.8_Mo_0.2_)O_3_ (ICSD-191551, α=β=γ=90°,a=b=c=3.9120 Å) and tetragonal SrMoO_4_ (ICSD-99089, α=β=γ=90°,a=b=5.3897 Å, c=12.0209 Å) structures. DPs were simulated via ‘ReciprOgraph’ software, developed by N. Schoeni and G. Chapuis.

(https://www.epfl.ch/schools/sb/research/iphys/teaching/crystallography/reciprograph/)

**Figure S7.** Comparison of reconstructed electron DPs from reduced SFM SOC lamella at different highlighted regions (1-4) with simulated electron DP using tetragonal Sr_3_FeMoO_7_ (RP phase) (ICSD-156787, α=β=γ=90°,a=b= 3.9120 Å, c=20.4667 Å) and cubic Sr_2_(Fe_1.33_Mo_0.66_)O_5.88_ (ICSD-168704, α=β=γ=90°,a=b=c=7.8677 Å) structures.

DPs were simulated via ‘ReciprOgraph’ software, developed by N. Schoeni and G. Chapuis.

(https://www.epfl.ch/schools/sb/research/iphys/teaching/crystallography/reciprograph/)
